# Validation of the German Version of the Social Functioning Scale (SFS) for Schizophrenia

**DOI:** 10.1371/journal.pone.0121807

**Published:** 2015-04-02

**Authors:** Jona R. Iffland, Denise Lockhofen, Harald Gruppe, Bernd Gallhofer, Gebhard Sammer, Bernd Hanewald

**Affiliations:** 1 Cognitive NeuroScience at the Centre for Psychiatry, Justus-Liebig-University, Giessen, Germany; 2 Bender Institute of Neuroimaging, Justus-Liebig-University, Giessen, Germany; UTHSCSH, UNITED STATES

## Abstract

Deficits in social functioning are a core symptom of schizophrenia and an important criterion for evaluating the success of treatment. However, there is little agreement regarding its measurement. A common, often cited instrument for assessing self-reported social functioning is the Social Functioning Scale (SFS). The study aimed to investigate the reliability and validity of the German translation. 101 patients suffering from schizophrenia (SZ) and 101 matched controls (C) (60 male / 41 female, 35.8 years in both groups) completed the German version. In addition, demographic, clinical, and functional data were collected. Internal consistency was investigated calculating Cronbach’s alpha for SFS full scale (α: .81) and all subscales (α: .59-.88). Significant bivariate correlation coefficients were found between all subscales as well as between all subscales and full scale (p <.01). For the total sample, principal component analysis gave evidence to prefer a single-factor solution (eigenvalue ≥ 1) accounting for 48.5 % of the variance. For the subsamples, a two-component solution (SZ; 57.0 %) and a three-component solution (C; 65.6 %) fitted best, respectively. For SZ and C, significant associations were found between SFS and external criteria. The main factor “group” emerged as being significant. C showed higher values on both subscales and full scale. The sensitivity of the SFS was examined using discriminant analysis. 86.5% of the participants could be categorized correctly to their actual group. The German translation of the SFS turned out to be a reliable and valid questionnaire comparable to the original English version. This is in line with Spanish and Norwegian translations of the SFS. Concluding, the German version of the SFS is well suited to become a useful and practicable instrument for the assessment of social functioning in both clinical practice and research. It accomplishes commonly used external assessment scales.

## Introduction

Deficits in social functioning are a well-studied core feature of schizophrenia [1,2]. Such deficits contain e.g. problems in community functioning and daily life, social and occupational functioning, residential maintenance, medication management and basic self-care [1,3]. Nowadays, they serve as an important outcome measure in studies exploring the disorder, its treatment and treatment success [2,4,5], especially, in studies examining the contribution of deficits in social functioning to risk of long term impairment [6]. Desirable therapeutic outcome should include both—maintained symptomatic remission, such as recovery from psychopathological symptoms (e.g. delusions and hallucinations) *and* appropriate social functioning [2,5,7]. Hence, researchers and clinicians agree that capturing psychopathological symptoms alone is not sufficient to reflect relevant outcomes. Therefore, social functioning is now commonly assessed in addition to symptomatology [6,8].

Despite the recent widespread use of the term, there is limited consensus even about the definition of social functioning. It is often used interchangeably with a variety of similar and overlapping concepts such as social performance, social adjustment, social dysfunction, social adaptation, social competence [1,2,5]. Social functioning is a heterogeneous concept comprising both differential societal roles in addition to actual social performance and patients may be differentially affected across these areas of functioning [8]. In addition, other factors like sex [9] might have an impact on social functioning.

As there is no consensus concerning social functioning, there cannot be a consensus regarding its measurement. Due to the absence of an objective gold standard, a multitude of different instruments are used to measure social functioning [1]. However, studies are limited. The assessment of social functioning might follow different strategies, like self-reports, informant reports, clinician ratings, and behavioural observation. Every strategy has its own pros and cons as well as its own meaningfulness in research projects (for an overview see [1]).

With the objective of aggregating different ways of measuring social functioning and identifying the best informant of patient functioning a board to validate everyday real-world outcomes (VALERO) was founded in 2007. In the context of the VALERO project 59 instruments measuring different constructs, like social functioning, quality of life, and everyday-living, were reviewed. Aspects as reliability, sensitivity to change, practicality and tolerability, usefulness for multiple informants, relationship with symptom measures and comprehensiveness of assessment were taken into account. Whereas the Heinrichs-Carpenter Quality-of-Life-Scale (QLS; [10]) scored most highly over all constructs, the Social Functioning Scale (SFS; [11]) and the Life Skills Profile (LSP; [12]) scored highest in their respective constructs (social functioning and everyday-living). These results indicate that there is not yet an entirely effective measure of functional outcome but that the current scales are viewed as useful and suitable in the interim [3]. According to Burns and Patrick [4] and Leifker and colleagues [3] the Social Functioning Scale (SFS) is one of the most cited and best rated (self-reporting) scales. It was constructed to measure those areas that are crucial to the community maintenance of individuals with schizophrenia. It was designed with respect to two requirements: (1) to provide a detailed assessment of patients’ strengths and weaknesses, both to guide an intervention and to provide the clinician with possible specific goals, and (2) the ability to synthesize such detailed reporting into coherent, reliable scales [11]. SFS scores are based on patients’ self-reports. The original scale has been demonstrated to be a reliable, valid and sensitive instrument for patients with schizophrenia [11], as have the two Spanish versions [13,14] and the Norwegian version [8] of the SFS. A German version of the SFS has not been published yet. The current study aimed to create a German version of the SFS, and to provide information about the reliability and validity of the translated questionnaire.

## Methods

### Sample

The study sample consisted of 101 participants with a diagnosis within the schizophrenia spectrum (schizophrenia (n = 92), schizoaffective disorder (n = 9)), and 101 control participants matched by age and sex. The clinical sample (SZ) was recruited from three Centres for Psychiatry in Hesse, Germany (Giessen [2], Bad Emstal), and consisted of 76 post-acute inpatients and 15 outpatients. Controls (C) participated in an online survey. They were students, staff members, and members of professorate of the University of Giessen and were contacted via mailing lists, therefore the controls were derived from a community sample. Patients were not included when matching at least one of the following exclusion criteria: mental retardation (IQ < 70), severe neurological disorder, acute self-endangering or endangering others, organic psychotic disorder, pharmaceutical or drug-induced psychotic disorder and being unable to sufficiently comprehend the German language. Control participants were excluded if they frequented a psychiatric and/or psychotherapeutic treatment during the last six months. Therefore, 74 of 795 participants that completed the online survey had to be excluded. The remaining participants were matched by gender and age to the 101 SZ participants. The study was approved by the Institutional Review Board of the University of Giessen and participants provided written informed consent before participating in the study. The declaration of Helsinki was conformed.

Demographic characteristics as well as group comparisons of both two study groups are shown in Table 1. The groups did not differ in age or sex. Matched controls were more likely to be engaged in a relationship, while almost 73% of the SZ were single. Controls showed higher levels of education as well as higher work status.

**Table 1:** Demographic characteristics and group comparisons for SZ and C .

	SZ (***n*** = 101)	C (***n*** = 101)	Group comparisons
**Sex (n, male / female)**	60 / 41	60 / 41	χ^2^_(2, n = 202)_ = .00, p = 1.0
**Age (in years)**	35.76 (10.04)	35.76 (10.09)	F_(1, 200)_ = .00, p = 1.0, ɳ^2^ = 0.00
**Duration of illness (in years)**	11.31 (9.08)	—	—
**Marital status**			
Single	73	29	χ^2^_(2, n = 202)_ = 42.0, p<.001
Life partner	22	40	
Married	6	32	
**Education type**			
Special school	1	1	FET, p<.001
Lower secondary education, no completion (ISCED Level 2^a^)	7	2	
Lower secondary education (ISCED Level 2^a^)	54	10	
Upper secondary education (ISCED Level 3A^a^)	35	88	
No graduation	4	0	
**Work status**			
Work / student, full time	7	87	FET, p<.001
Work part time	10	13	
Work occasionally	3	0	
Housekeeping	2	0	
Vocational training	10	0	
Unemployment	31	1	
Disability pension	19	0	
Sheltered workplace	19	0	
**Housing**			
Living independently / with partner	50	92	FET, p<.001
Living with parents / relatives	17	9	
Institutionalized	14	0	
Homeless	1	0	
Unknown	19	0	

N or means (SD) are reported. Chi-square analyses (χ^2^) or Fisher’s exact tests (FET) for categorical data; ANOVAs (F) and eta-correlation coefficients (ɳ^2^) for continuous data are reported.

^a^[36]

### Clinical Assessment

Diagnoses were based on the Structured Clinical Interview for DSM-IV Axis I disorders [15] and available medical records.

The *Positive and Negative Syndrome Scale* (PANSS; [16]) and the *Clinical Global Impression Score* (CGI; [17]) were used to assess symptom severity. For the sample of patients, measures of social functioning included the *Global Assessment of Functioning* (GAF; [18]) and the *Disability Assessment Schedule* (DAS-M; [19]). For the community sample, measures of social functioning included the full version of the *WHO Disability Assessment Schedule 2*.*0* (WHODAS 2.0; [20]) and the *Soziale Aktivität Selbstbeurteilungs-Skala* (SASS; [21]). For both groups, personal data on sex, age, occupational status, education type and living situation were collected. Diagnostic assessment was performed by trained 5 psychiatrists and clinical psychologists who are trained in rating the applied clinical scales. The success of the training was verified by computing intraclass correlation coefficients (ICC) for interviews and tutorial videos (ICC_(3,k)_ = .92; 95% CI [.87,.96]).

Values for clinical symptoms and acquired functioning scores are shown in Table 2. Patients suffering from schizophrenia showed in average minimal levels of positive symptoms and mild levels of negative symptoms. Nevertheless, a rather high impairment of social functioning, which was operationalized using the GAF scale (average score of 52 out of 100), was found. Similar to this result, the DAS-M score showed an average score of 2.8, indicating a low level of social adaption if compared to general population. The CGI score showed a moderate to marked severity of illness. Matched controls scored low in the WHODAS 2.0 (range 36–180, simple scoring). The SASS values remained within the normal range (35–52), indicating no impairment of social functioning.

**Table 2:** Symptom loads and Functioning Scores.

	SZ (***n*** = 101)	C (***n*** = 101)
**PANSS total**	66.0 (20.7)	—
**PANSS positive**	12.9 (5.3)0	—
**PANSS negative**	18.8 (6.9)0	—
**PANSS general**	34.3 (11.5)	—
**GAF**	52.0 (12.3)	—
**DAS-M**	2.8 (0.8)	—
**CGI 1**	4.3 (1.0)	—
**WHODAS 2.0**	—	45.1 (14.0)
**SASS**	—	41.0 (5.2)0

Means (SD) are presented.

### The Social Functioning Scale

The Social Functioning Scale (SFS) was developed to assess social skills and performance, and to cover functions that are of importance for patients suffering from schizophrenia. Therefore, the SFS reflects the areas focused on in various psychosocial intervention programs [22–24] as well as the disabilities and the impairments assessed by the Disability Assessment Schedule [25]. The SFS represents both strengths and weaknesses of the patients in order to facilitate the planning of interventions and individual goals [11]. The SFS assesses the presence or absence of key skills and social behaviours in the individual. The SFS also distinguishes lack of competence from lack of performance. Lack of competence refers to the absence or loss of a skill, while lack of performance refers to non-use of an available skill [11].

The SFS is a self-administered questionnaire and consists of 76 items with varying response formats. Four items are dichotomous questions, one item records the time of getting up, one item is rated on a three-point Likert scale, two items are rated on a five-point Likert scale, and 68 items are rated on a four-point Likert scale of frequency or ability. A higher score indicates more competent behaviour or higher frequency. All items are assigned to seven subscales. Each subscale score is the sum of all item values of that subscale. Every subscale value is standardized and normalized to a *Scaled Score* (Mean = 100, SD = 15), based on a sample of 334 individuals with schizophrenia [11]. The SFS full scale score is computed as the mean of the seven subscales scaled scores. The seven subscales are: (1) social engagement/withdrawal (time spent alone, initiation of conversation, social avoidance) [*withdrawal*]; (2) interpersonal behaviour (number of friends/having a romantic partner, quality of communication) [*interpersonal*]; (3) pro-social activities (engagement in a range of common social activities, e.g. sport) [*pro-social*]; (4) recreation (engagement in a range of common hobbies, interests, pastimes etc.) [*recreation*]; (5) independence-competence (ability to perform skills necessary for independent living) [*independ-comp*]; (6) independence-performance (performance of skills necessary for independent living) [*independ-perf*]; (7) employment/occupation (engagement in productive employment or a structured program of daily activity) [*employment*].

The German version of the SFS was created in 2010 by two of the authors (JRI, BH). In order to monitor the quality of the translation, both the German version and the English version were administered to 31 students of English language at Master level in a test-retest-design. The test-retest-interval was one week, the order of the two versions was permuted (German—English, n = 12; English—German, n = 19). The rank stabilities indicated by Spearman rho coefficients have been satisfying for all subscales (from. 73 to. 90 and. 87 for the SFS full scale) except for the subscale (2) interpersonal (Spearman rho = .43). This low estimate could be a result of the small number of items (n = 3). The stability of means assessed by paired t-tests revealed no significant mean difference for any scale.

### Data Analyses and Statistics

Data analyses followed the work of Hellvin and colleagues [8] using the statistical software package IBM SPSS Statistics Version 22 [26]. Demographic data were analyzed using chi-square (χ^2^) or Fisher’s exact tests as appropriate (categorical data) and analyses of variance (ANOVAs) (continuous data). Squared eta-correlation coefficients (ɳ^2^) refer to effect sizes. Reliability analyses were carried out by enumerating mean item-total correlations, mean inter-item correlations, and Cronbach’s alpha for the seven subscale scores and the full scale score. Pearson’s product-moment correlation coefficients (*r*) between the SFS full scale score and the seven subscale scores as well as between the seven subscale scores were reported. For both groups, the relationships between the SFS score, the GAF score, and key demographic, clinical, and functional characteristics were calculated applying bivariate Pearson’s product-moment correlation coefficients (*r*; age, symptom loads, functioning scores, point-biserial correlation coefficients (r_pb_; sex), and one-way ANOVAs to reveal differences between the categories (marital status, education, work status, housing). Principal component analyses with Varimax rotation (eigenvalues ≥ 1.0) were performed using the seven subscale scores for the total sample as well as within the SZ and C groups separately. *Sex* and *group* differences in SFS subcale scores were analyzed by an overall multivariate analysis (MANOVA) with the two independent between-subjects factors *group* and *sex*. In case of significant overall effects, multiple two-way ANOVAs were conducted. For the SFS full scale score, a 2 x 2 ANOVA with the two independent factors group and sex was computed. Effect sizes are reported as the squared eta-correlation (ɳ^2^). A discriminant analysis was performed for the prediction of group membership. Discriminant analysis was based on the set of seven SFS subscale scores, classifying participants in the C group from the clinical group. In order to identify the underlying differences between correctly and false negatively assigned patients a set of ANOVAs was performed for SFS scores, key demographic, clinical and functional characteristics. Again, effect sizes were reported by the squared eta-correlation (ɳ^2^). To investigate floor and ceiling effects a frequency analysis was carried out.

## Results

### Reliability

Reliability measures for the SFS representing measures of internal consistency are presented in Table 3. Cronbach´s alpha for the six subscales (1) withdrawal, (2) interpersonal, (3) pro-social, (4) recreation, (5) independ-comp and (6) independ-perf ranged from. 59 to. 88 with a score of. 81 for the full scale. Mean item-total correlation coefficients (r) ranged from. 23 to. 58 for the six subscales and. 56 for the SFS full scale. Furthermore, mean inter-item correlation (r) ranged from. 09 to. 38 for the six subscales and. 40 for the SFS full scale. Subscale (7) employment contains a filter item (employment yes/no) with different subsequent items, therefore, measures of internal consistency were not computed.

**Table 3:** Reliability measures for the Social Functioning Scale, German version.

		(1)	(2)	(3)	(4)	(5)	(6)	(7)
	Full scale	withdrawal	interpersonal	pro-social	recreation	independ-comp	independ-perf	employment
**No. of Items (n)**	7	5	3	22	15	13	13	-
**Mean item-total correlation (r)**	.56	.35	.41	.35	.23	.58	.47	-
**Mean inter-item correlation (r)**	.40	.21	.34	.15	.09	.38	.28	-
**Cronbach’s alpha**	.81	.59	.60	.79	.60	.88	.81	-

Results are reported for total sample (*n* = 202).

### Validity

Table 4 shows bivariate correlation coefficients between the full scale score and the seven subscales scores for the total sample. All correlation coefficients are higher than *r* >. 61, thus supporting previous considerations that the scale may be represented by a single composite score [11]. The intercorrelation patterns for the three subscales (2) interpersonal, (6) independence-competence, and (7) employment are somewhat lower.

**Table 4:** Bivariate correlation coefficients between Social Functioning Scale (SFS) full scale and subscale scores (*n* = 202).

		(1)	(2)	(3)	(4)	(5)	(6)
	Full scale	withdrawal	interpersonal	pro-social	recreation	independ-comp	independ-perf
**(1) withdrawal**	.72						
**(2) interpersonal**	.69	.54					
**(3) pro-social**	.76	.48	.38				
**(4) recreation**	.71	.42	.29	.64			
**(5) independ-comp**	.64	.33	.32	.34	.31		
**(6) independ-perf**	.72	.40	.29	.46	.52	.55	
**(7) employment**	.61	.32	.28	.39	.30	.37	.41

For all correlations: p <. 01.

For the patient sample (SZ) (see Table 5), the SFS full scale score correlated highly significant with the GAF (r = .46, p <. 001) and CGI1 (r = -.45, p <. 001). A significant correlation was found between the SFS full scale score and the observed DAS-M score (r = -.43, p <. 001). High DAS-M score indicates lower social functioning. Moderate correlations were found between the SFS full scale score and clinical symptoms (PANSS total score: r = -.31, p <. 01, PANSS positive subscale score: r = -.33, p <. 01, PANSS negative subscale score: r = -.36, p <. 001, PANSS general subscale score: r = -.20, p <. 05). All correlations between GAF score and clinical symptoms and functioning scores were highly significant (PANSS total score: r = -.68, p <. 001, PANSS positive subscale score: r = -.53, p <. 001, PANSS negative subscale score: r = -.61, p <. 001, PANSS general subscale score: r = -.61, p <. 001, CGI1: r = -.80, p <. 001, DAS-M: r = -.84, p <. 001). For the patient sample (SZ), neither sex nor age had effects on the SFS full scale score and the GAF Score. For the SFS full scale score, both marital status and work status significantly differed between the categories. For marital status, mean values increased over *Single < Life partner < Married*, for work status, mean values increased over *Disability pension < Work full time < Unemployment < Sheltered workplace < Housekeeping < Work part time < Vocational training < Work occasionally*. Analogously, for the GAF score, education, work status, and housing significantly differed between the categories. For education, mean values increased over *Special school < ISCED Level 2* ^*a*^, *no completion < No graduation < ISCED Level 2* ^*a*^ *< ISCED Level 3* ^*a*^, for work status, the order of mean values was: *Disability pension < Unemployment < Housekeeping < Sheltered workplace < Work occasionally < Vocational training < Work full time < Work part time*. For housing, mean values increased over *Homeless < Institutionalized < Living independently / with partner < Living with parents / relatives*. In the C group, a highly significant correlation between SFS full scale and sex (r_pb_ = .46, p <. 001) was found. Additionally, the C group significantly differed between the housing categories (*Living with parents / relatives < Living independently / with partner*). Highly significant correlations were found between the SFS full scale score and functioning scores (WHODAS 2.0: r = -.46, p <. 001, SASS: r = .47, p <. 001) (Table 5).

**Table 5:** Associations of the Social Functioning Scale (SFS) full scale score and the GAF score with demographic characteristics, symptom load and functional measures for SZ and C.

	SZ (***n*** = 101)		C (***n*** = 101)
	SFS	GAF	SFS
**Demographics**			
Sex	-.17***	-.10***	-.46***
Age	-.01***	-.04***	-.02***
Marital status	F_(2, 98)_ = 6.15**	F_(2, 98)_ = 0.31	F_(2, 98)_ = 1.66
	ɳ^2^ = 0.11	ɳ^2^ = 0.01	ɳ^2^ = 0.03
Education	F_(4, 96)_ = 0.90	F_(4, 96)_ = 2.82*	F_(2, 98)_ = 1.25
	ɳ^2^ = 0.04	ɳ^2^ = 0.11	ɳ^2^ = 0.04
Work status	F_(7, 93)_ = 3.42**	F_(7, 93)_ = 3.31**	F_(2, 98)_ = 0.47
	ɳ^2^ = 0.21	ɳ^2^ = 0.20	ɳ^2^ = 0.01
Housing	F_(3, 97)_ = 2.12	F_(3, 97)_ = 3.76*	F_(2, 98)_ = 4.00*
	ɳ^2^ = 0.08	ɳ^2^ = 0.13	ɳ^2^ = 0.04
**Symptom load**			
PANSS total	-.31**	-.68***	—
PANSS positive	-.33**	-.53***	—
PANSS negative	-.36***	-.61***	—
PANSS general	-.20*	-.61***	—
**Functioning**			
GAF	.46***	—	—
DAS-M	-.43***	-.84***	—
CGI1	-.45***	-.80 ***	—
WHODAS 2.0	—	—	-.46***
SASS	—	—	.47***

Point-biserial correlation coefficients (r_pb_) are presented for sex. Pearson’s product-moment correlation coefficients (r) are presented for age, symptom loads, and functioning. ANOVAs (F) and eta-correlation coefficients (ɳ^2^) for marital status, education, work status, and housing

*p<.05;

**p<.01;

***p<.001

### Principal component analyses

The results for the exploratory principal component analyses are shown in Table 6. A one-factor solution was found for the total sample. The subscale (7) employment showed the lowest loading. The first unrotated component accounted for 48.5% of the variance. For the clinical sample, a two-component solution was found. Both factors (eigenvalue ≥ 1) accounted for 57% of the variance in total. The first unrotated component on its own explained 41.2% of the variance. The first rotated component consisted of the four subscales (1) withdrawal, (2) interpersonal, (3) pro-social and (4) recreation. The second rotated component is composed of the three subscales (5) independ-comp, (6) independ-perf and (7) employment.

**Table 6:** Principal Component analyses of seven Social Functioning Scale (SFS) subscales.

	Total sample	SZ			C			
	(***n*** = 202)	(***n*** = 101)			(***n*** = 101)			
	Unrotated	Unrotated	Rotated		Unrotated	Rotated		
	F1	F1	F1	F2	F1	F1	F2	F3
**(1) withdrawal**	.72	.65	**.78**	.01	.64	**.76**	.08	.25
**(2) interpersonal**	.62	.55	**.66**	.01	.57	**.76**	.00	.19
**(3) pro-social**	.77	.78	**.73**	.32	.52	.18	-.08	**.81**
**(4) recreation**	.73	.74	**.69**	.30	.66	.03	.36	**.77**
**(5) independ-comp**	.65	.54	.20	**.66**	.48	.07	**.81**	-.03
**(6) independ-perf**	.75	.77	.44	**.73**	.62	.09	**.84**	-.18
**(7) employment**	.62	.35	-.09	**.74**	-.26	-.6	-.16	**.33**
**eigenvalue**	3.4	2.9	2.3	1.7	2.1	1.6	1.5	1.5
**% variance explained**	48.5	41.2	32.8	24.2	30.6	22.3	21.8	21.4
**cum. % variance explained**	48.5			57.0			44.2	65.6

Reported are factor loadings from the unrotated first component, and factor loadings from the rotated components when more than one was indicated (eigenvalue > 1.0, Varimax rotation).

For the C group, three components with eigenvalue ≥ 1 were found. The first unrotated component explained 30.6% of the variance. The three rotated components accounted for 65.6% of the variance in total. The first rotated component consisted of the two subscales (1) withdrawal and (2) interpersonal. The second rotated component is composed of the two subscales (5) independ-comp and (6) independ-perf, while (3) pro-social, (4) recreation, and (7) employment constituted the third rotated component.

### Group comparisons

**Fig 1:**
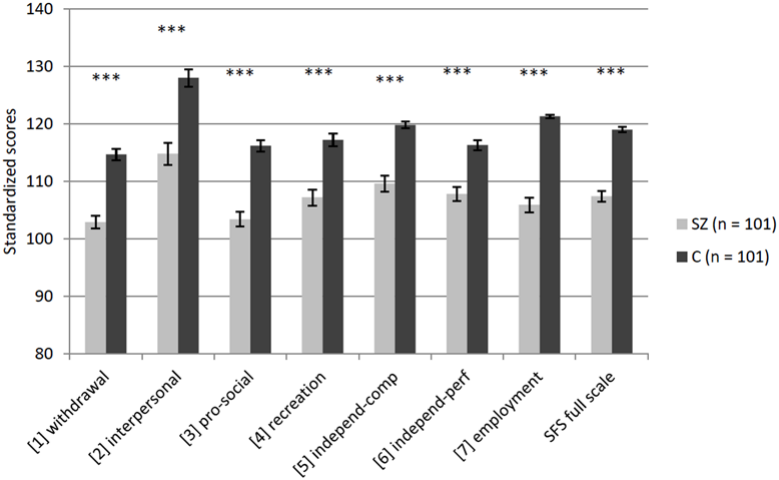
Group comparisons of standardized mean scores on the Social Functioning Scale for SZ and C. Error bars indicate the standard error of the mean (*p<.05; **p<.01; ***p<.001).

The multivariate analysis of variance for the 7 SFS subscales showed significant effects of the factor *group* F_(7, 192)_ = 30.91, p<.001, Pillai’s Trace = .530, partial ɳ^2^ = 0.53, and the factor *sex* F_(7, 192)_ = 6.80, p<.001, Pillai’s Trace = .199, partial ɳ^2^ = 0.20, but not for the *group x sex* interaction F_(7, 192)_ = 0.58, p = .77, Pillai’s Trace = .021, partial ɳ^2^ = 0.02. Results of the univariate analyses of variance for each SFS subscale and the SFS full scale score are shown in Table 7. There were significant *group* differences on all subscales as well as on the full scale score: participants with SZ scored significantly poorer than the matched controls (Fig 1).

**Table 7:** Group comparisons of standardized mean scores on the Social Functioning Scale for SZ and C separated by *group* and *sex*.

		M (SD)									
		SZ		C		ANOVA					
	range (theoretical)	Male (***n*** = 60)	Female (***n*** = 41)	Male (***n*** = 60)	Female (***n*** = 41)	***Group*** F(1,198)	Effect size (ɳ^2^)	***Sex*** F(1,198)	Effect size (ɳ^2^)	***G x S*** F(1,198)	Effect size (ɳ^2^)
**(1) withdrawal**	57.5–133.0	100.8 (10.4)	105.9 (10.7)	111.3 (11.0)	119.8 (7.2)	070.38***	0.26	22.34***	0.10	1.34	<0.01
**(2) interpersonal**	55–145	112.8 (18.2)	117.7 (20.8)	124.6 (15.2)	132.9 (13.3)	030.55***	0.13	07.21**	0.04	0.48	<0.01
**(3) pro-social**	65–145	103.3 (13.2)	103.6 (13.0)	114.2 (10.8)	119.1 (9.4)	061.04***	0.24	02.33	0.01	1.88	<0.01
**(4) recreation**	57–145	107.1 (14.2)	107.4 (13.8)	114.8 (12.3)	120.3 (8.4)	032.72***	0.14	02.60	0.01	1.98	0.01
**(5) independ-comp**	49–123	108.5 (14.2)	111.3 (13.7)	119.2 (6.3)	120.7 (5.8)	042.18***	0.18	02.01	0.01	0.18	<0.01
**(6) independ-perf**	53–131	104.9 (10.8)	112.1 (11.5)	113.5 (9.0)	120.3 (6.8)	036.33***	0.16	25.42***	0.11	0.03	<0.01
**(7) employment**	81.5–122.5	106.2 (12.6)	105.6 (13.7)	121.7 (2.1)	120.6 (3.0)	128.53***	0.39	00.39	<0.01	0.05	<0.01
**SFS full scale**	59.7–134.9	106.2 (8.8)	109.1 (8.0)	117.0 (5.3)	121.9 (3.8)	144.94***	0.42	15.70***	0.07	1.05	<0.01

Means (M) and standard deviations (SD) separated by *group* and *sex* are presented. Univariate ANOVAs (F) and eta-correlation coefficients (ɳ2) for the effects of *group*, *sex*, and the interaction *group x sex* (*G x S*) are reported.

**p<.01;

***p<.001

Significant *sex* differences were found on the three subscales (1) withdrawal, (2) interpersonal, and (6) independ-perf, and on the SFS full scale. Due to the significant correlation coefficient between sex and SFS full scale in the group of matched controls, ANOVAs for the subscales and the full scale were separately computed for the factor *sex* within each group. For the group of matched controls significant sex differences were found on the full scale (F_(1.99)_ = 16.13, p<.001, ɳ^2^ = 0.21) and all subscales ((1) withdrawal: F_(1.99)_ = 18.95, p<.001, ɳ^2^ = 0.16, (2) interpersonal: F_(1.99)_ = 7.90, p<.01, ɳ^2^ = 0.07, (3) pro-social: F_(1.99)_ = 5.50, p<.05, ɳ^2^ = 0.05, (4) recreation: F_(1.99)_ = 6.10, p<.05, ɳ^2^ = 0.06, (6) independ-perf: F_(1.99)_ = 16.94, p<.001, ɳ^2^ = 0.15, (7) employment: F_(1.99)_ = 5.10, p<.05, ɳ^2^ = 0.05) except on the subscale (5) independ-comp, whereas for the sample of patients significant sex differences were only found on the subscales (1) withdrawal (F_(1.99)_ = 5.85, p<.05, ɳ^2^ = 0.06) and (6) independ-perf (F_(1.99)_ = 16.94, p<.001, ɳ^2^ = 0.10). Except on subscale (7) employment, on all listed subscales and on the full scale, females scored significantly higher in each group. On the subscale (7) employment, C males showed significantly higher scores compared to C females.

### Discriminant analysis

Using discriminant analysis 77% of the SZ group and 96% of the C group could be correctly assigned (Table 8).

**Table 8:** Discriminant analysis of the seven SFS subscales.

		Predicted group		
		SZ	C	Total
**Actual group**	**SZ**	78	23	101
	**C**	4	97	101
	**Total**	82	120	202

Following Birchwood et al. (1990) [11], Torres et al. (2005) [14], and Hellvin et al. (2010) [8] a frequency analysis was performed regarding the distribution of scores for both group. The median of the SZ group was in the score range of 106–115 (Md = 107.1) and the scores were more widely scattered than the scores of the C group, clustering around a higher median (Md = 119.1). Only 1 participant of the SZ sample, but 10 participants of the C sample scored in the highest score range. None of the C group, but 45 participants of the SZ group scored in the two lowest score ranges (Table 9).

**Table 9:** Distribution of scores on the Social Functioning Scale (SFS) full scale.

	SZ (n = 101)		C (n = 101)	
SFS full scale score	N	Cum %	N	Cum %
85–95	8	7.92%	0	
96–105	37	44.55%	0	
106–115	42	86.13%	24	23.76%
116–125	13	99.01%	67	90.10%
126–135	1	100%	10	100%

Based on the data of the discriminant analysis the 23 patients assigned to the community sample and the remaining 78 correctly assigned patients were compared regarding demographic and psychopathological characteristics as well as SFS full scale and the 7 subscales (Table 10). For the two groups, no sex and age differences were found. The false negatively assigned patients scored significantly lower on all psychopathological scales such as PANSS and CGI, but significantly higher on SFS full scale and all SFS subscales. They also showed significantly lower impairment on GAF and CGI (Table 10).

**Table 10:** Group comparisons of correctly (CA) and false negatively (FNA) assigned patients.

	CA (n = 78)	FNA (n = 23)	Group comparisons
**Sex (n, male/female)**	47/31	13/10	χ^2^_(2, n = 202)_ = .10 p = .75
**Age**	35.7 (10.5)	35.9 (8.6)	F_(1.100)_ = .0 n.s., ɳ^2^ = .00
**(1) withdrawal**	99.9 (9.4)	112.6 (10.0)	F_(1.100)_ = 31.1 p<.001, ɳ^2^ = 0.24
**(2) interpersonal**	111.6 (19.4)	125.5 (15.1)	F_(1.100)_ = 10.0 p<.01, ɳ^2^ = 0.09
**(3) pro-social**	99.8 (11.4)	115.7 (10.9)	F_(1.100)_ = 35.1 p<.001, ɳ^2^ = 0.26
**(4) recreation**	105.5 (13.1)	113.1 (15.7)	F_(1.100)_ = 5.4 p<.05, ɳ^2^ = 0.05
**(5) independ-comp**	107.6 (14.8)	116.6 (7.9)	F_(1.100)_ = 7.9 p<.01, ɳ^2^ = 0.07
**(6) independ-perf**	106.0 (11.6)	114.0 (9.3)	F_(1.100)_ = 9.3 p<.01, ɳ^2^ = 0.09
**(7) employment**	102.9 (13.0)	116.4 (5.4)	F_(1.100)_ = 23.5 p<.001, ɳ^2^ = 0.19
**SFS full scale**	104.8 (7.4)	116.3 (5.8)	F_(1.100)_ = 46.7 p<.001, ɳ^2^ = 0.32
**PANSS total**	69.7 (20.7)	54.0 (15.4)	F_(1.100)_ = 11.3 p<.001, ɳ^2^ = 0.10
**PANSS positive**	13.7 (5.4)	10.5 (4.2)	F_(1.100)_ = 6.8 p<.05, ɳ^2^ = 0.07
**PANSS negative**	20.2 (6.8)	14.4 (5.4)	F_(1.100)_ = 13.9 p<.001, ɳ^2^ = 0.12
**PANSS general**	35.9 (11.7)	29.1 (9.3)	F_(1.100)_ = 6.4 p<.05, ɳ^2^ = 0.06
**GAF**	49.8 (10.9)	60.3 (13.2)	F_(1.100)_ = 14.8 p<.001, ɳ^2^ = 0.13
**DAS-M general**	2.9 (0.8)	2.3 (0.9)	F_(1.100)_ = 9.6 p<.01, ɳ^2^ = 0.09
**CGI 1**	4.5 (0.9)	3.7 (0.9)	F_(1.100)_ = 14.1 p<.001, ɳ^2^ = 0.13

Means (SD) are presented. Chi-square analyses (χ2) for categorical data; ANOVAs (F) and eta-correlation coefficients (ɳ2) for continuous data are reported.

## Discussion

The aim of the study was to produce a German translation of the Social Functioning Scale (SFS) and to investigate its reliability and validity. The results indicate that the German translation of the SFS complies with the obligatory test criteria.

Due to our matching-procedure groups were balanced concerning age and sex. Compared to a community sample, participants with SZ were significantly more frequent single, while normal participants significantly more often lived with a partner or were married. Furthermore, patients reported a lower educational status and a poorer working status. Deficits in social relations and employment status are frequently reported in patients suffering from schizophrenia and are therefore often highlighted as a treatment target [27,28]. In order to validate our matching-procedure and due to the differences between the included groups regarding demographic characteristics, we examined the relationships between the SFS and marital status, education, housing and work status. For the SZ participants, we found significant relationships between marital status and work status and SFS indicating that employment and stable relationships come along with higher social functioning. For the matched controls, we found no significant associations between SFS and work status, marital status, and education, respectively, but a weak significant association was found between the SFS and housing. However, due to ceiling effects significant correlations were not expected in the group of matched controls. All in all, analyses suggested that marital status, education, housing, and work status do not play a decisive role in explaining the observed differences in social functioning between the two samples.

### Reliability

Examining the internal consistencies of the subscales, Cronbach’s alpha values were in the range from. 59 (poor) to. 88 (good). The lowest estimate (.59; (1) withdrawal) could be a result of the small number of items in this subscale (n = 5), hence, it is likely a bandwidth-fidelity dilemma (i.e. small number of items, and, associated therewith, diversity (bandwidth) of items, lead to a reduced reliability score). Enlargement of the scale is usually proposed to solve the problem. For the full scale, Cronbach’s alpha of. 81 indicates a good reliability of the German translation. These results are comparable to the findings of the original English scale, as well as both the Spanish and the Norwegian translations, but the scores are somewhat lower (Table 11). Item-total correlations as well as inter-item correlations show a comparable pattern to the original English sample, to the Spanish samples and to the Norwegian sample, but on a slightly lower level with variations above and below. The various structures of the subscales, e.g. number of items, response options etc. may account for the obvious heterogeneity of the psychometric properties in the seven subscales.

**Table 11:** Reliability measures of the English version, Spanish versions, and Norwegian version for the Social Functioning Scale (if reported).

		(1)	(2)	(3)	(4)	(5)	(6)	(7)
	Full scale	withdrawal	interpersonal	pro-social	recreation	independ-comp	independ-perf	employment
***English version***								
**Birchwood et al. (1990), n = 434**								
Mean item-total correlation (r)	.71	.49	.40	.37	.30	.55	.53	-
Mean inter-item correlation (r)	.44	.37	.36	.29	.25	.35	.33	-
Cronbach’s alpha	.80	.72	.71	.82	.69	.87	.85	-
***Spanish versions***								
**Vázquez Morejón & Jiménez G-Bóveda (2000), n = 150**								
Mean item-total correlation (r)	.68	.41	.35	.43	.28	.62	.52	-
Cronbach’s alpha	.85	.66	.45	.86	.67	.90	.86	-
**Torres & Olivares (2005), n = 205**								
Mean item-total correlation (r)	-	.57	.67	.84	.85	.46	.59	.56
Cronbach’s alpha	-	.80	.80	.69	.74	.79	.77	.80
***Norwegian version***								
**Hellvin et al. (2010), n = 300**								
Mean item-total correlation (r)	.66	.44	.45	.46	.36	.51	.50	.40
Mean inter-item correlation (r)	.51	.22	.37	.25	.17	.31	.30	.19
Cronbach’s alpha	.81	.68	.63	.88	.76	.82	.83	.60

### Validity

Significant inter-correlations were found between the subscales, but also between the subscales and the full scale at a moderate level. The results are comparable with the findings of Birchwood et al. (1990) [11]for the English version of the Social Function Scale as well as for the results of Hellvin et al. (2010) [8]for the Norwegian version. The inter-correlation coefficients found by Hellvin et al. (2010) were slightly higher. The inter-correlation pattern suggests that the subscales are connected by a common construct, each subscale representing different aspects of the construct ‘social functioning’.

External criteria such as the functioning scores like GAF, CGI, and DAS-M correlated significantly with the SFS in the expected direction, indicating that the patients´ self-reports and the clinician-based ratings share a moderate degree of variance. This fact supports the assumption that the SFS can be seen as a valid measure of social functioning. However, there is no complete redundancy between the SFS scores and the clinician-based ratings, indicating that the SFS represents the subjective and individual view of one’s life, providing additional independent and useful information to the view of the clinicians. Furthermore, the mentioned significant relationship between GAF rated by clinician and self-reported social functioning contrasts with reports that stated that in this population reduced insight is often present [29–31]. Another aspect could be that even if a person’s insight is reduced, the subjective feelings by definition are an important factor of subjective quality of life and social functioning. Compared to the results of Vázquez Morejón & Jiménez G-Bóveda (2000) [13]and Hellvin and colleagues (2010) we found a slightly smaller relationship between SFS and GAF but heading in the same direction.

Similar to the findings of Hellvin et al. (2010), we found significant negative associations between the PANSS total score and all PANSS subscales, respectively, and the SFS full scale score. Such associations between functional outcome and negative symptoms [32,33], and positive symptoms [34,35] have been reported before.

In our study, the relationships between SFS full scale score and psychopathological parameters (PANSS) were lower than between GAF and psychopathological and demographic parameters. It should be noted that both GAF and PANSS are ratings conducted by the same trained clinicians. This increases the intra-rater reliability of ratings. Additionally, while assessing the GAF score the clinician gathers information about the patient, e.g. work status, education, marital status, and symptom severity, and takes them into account. Therefore, higher associations between the GAF score and demographic parameters could be expected.

External criteria for the community sample were the WHODAS 2.0 and the SASS. The associations between SFS and WHODAS 2.0 and SASS, respectively, were at a moderate level. Again, these data emphasize the assumption that the SFS can be seen as a valid measure of social functioning.

#### Principal component analyses

In line with Hellvin et al. (2010), the principal component analyses produced a two component solution for the SZ group and a three component solution for the matched controls. The accounted variance of 57% (SZ) and 65.6% (C), respectively, was satisfactory and comparable to the findings of Hellvin et al. (2010) considering the community sample and the participants with SZ. Compared to the results of Birchwood et al. (1990) the accounted variance was higher for both the SZ participants and the matched controls.

The factorial structure did not substantially differ from that reported by Hellvin et al. (2010). For the sample of patients, Hellvin and colleagues (2010) found two factors as well. The first factor consisted of the subscales (1)–(6), and the second factor consisted of the subscale (7) employment solely. In the present study, the second factor was increased by the ‘independence’ cluster. And for the community sample, there was just one single change in the factorial structure compared to Hellvin et al. (2010). The subscale (4) recreation changed its position in the factor solution forming a new factor consisting of (4) recreation, (3) pro-social activities and (7) employment. In our opinion, these new composed factor solutions are highly reasonable. Especially for participants with SZ—only 20 patients were employed—the relationship between employment, independency, and autonomy plays a crucial role. For the community sample, we found three factors composed of subscales that are thematically related. The first factor represents aspects of social engagement and interpersonal communication, the second factor consists of the ‘independence’ cluster, and the third factor combines recreation behaviour and to a smaller extent—due to ceiling effects—employment status.

### Group comparisons

In line with Birchwood et al. (1990) and Hellvin et al. (2010), the SZ participants scored significantly poorer on all subscales and on the SFS full scale score compared to matched controls indicating that the German version of the SFS assesses social functioning comparable to the English and Norwegian version. These results can be seen as further evidence for the good reliability and validity of the German translation. Additionally, we detected a relationship between SFS and sex, especially for the community sample. This is also in line with a prior study [9]. We found that C females scored significantly higher on all subscales except for the subscales independence-competence and employment. There was no significant sex difference in the subscale independence-competence, and C males scored significantly higher on the subscale employment.

This could be due to a genuinely higher level of social functioning in women, otherwise the SFS may detect aspects of social functioning levels which are more strongly represented in women than in men. Nevertheless, men just showed a higher level of social functioning on the subscale employment. On the one hand, this might indicate that the profession may constitute a significant proportion of life in men, and men may define their self-image more about the profession than women do. On the other hand, it could be due to the fact that women are more likely than men to work part time or to work at home taking care of the family. In this case, the SFS subscale employment would automatically be lowered by one raw point, reflecting rather sociological than psychological issues.

It is noteworthy that gender differences were also found in patients, but significant differences were found for the subscales withdrawal and independence-performance only. In the relevant literature the number of studies on sex differences in social functioning is small. However, there is clear evidence for sex differences in neurocognitive performance which is linked to social functioning [9]. It will be of interest to put more emphasis on this issue. By way of qualifying that, it must be noticed that the SFS was developed to capture the social functioning level of people suffering from schizophrenia, but not for a community sample.

### Discriminant analysis

The SFS scale discriminates between control participants and participants with SZ, recognizable by significantly different mean scores for all scales as well as by the distribution of the SFS full scale scores. Regarding the discriminant analysis, the SFS provides a satisfying sensitivity, as 23% of the patients were false negatively assigned as control participants (type 2 error), and a very high specificity. Only 3.9% of the community sample was assigned false positively as patients (type 1 error).

Overall, the discriminant analysis showed 86.5% correct results. These results show distinct differences between SZ and C in the social functioning level. The SFS is not considered to be a screening instrument for schizophrenia, and therefore, the results of the discriminant analysis can be regarded as satisfactory. Within the group of participants with SZ the SFS distinguishes different functional levels, hence, reflecting the illness-related heterogeneity of social functioning. In contrast, the scattering is lower in control participants, and can be understood by ceiling effects.

The amount of type 2 error demonstrates that suffering from schizophrenia does not necessarily imply a poor level of social functioning. The false negatives identified as belonging to the community sample showed on average a significant higher level of social functioning in all subscales of the SFS, compared to the correctly assigned patients. In addition, the group of false negatively patients differed significantly from the remaining sample of patients with regard to clinician’s ratings such as GAF, CGI and PANSS, but there were no differences in sex and age. These results indicate that there are fewer psychopathological abnormalities in patients with higher social functioning levels. Higher social functioning levels indicate less impairment related to illness, measured with the CGI and the GAF. Higher social functioning level in a subset of the sample of patients is not attributable to specific subscales, but is evident in all subscales of the SFS.

### Limitations

This study has several limitations. It is unclear whether GAF, DAS-M or CGI can be regarded as valid external criteria for self-rated social functioning because scoring by trained clinicians does not necessarily reflect the real social skills of observed patients. Rater biases may be present.

Anyhow, with all the uncertainty of the underlying construct it seemed to be legitimate to use established scales to assess social functioning levels of patients or control participants, respectively, and to put them into relationship to the SFS.

Like Birchwood et al. (1990) and Hellvin et al. (2010) we found low inter-item correlations as well as satisfactory scores of Cronbach’s alphas. These low inter-item-correlations might indicate a constraint of the SFS although their relevance remains uncertain, given satisfactory alpha scores.

An additional restriction might be that no official back translation was conducted to identify and remove potential inconsistencies between the English and German version. Another limitation might be the fact that we used different types of measurement, online survey (C) vs. paper-pencil (SZ). This questions the comparability of the scores of both groups. However, our results for C regarding scoring and factor structure are comparable to those of Hellvin pointing to a limited impact of the used types of measurement. Therefore, our data seem to be commensurable for the C as well and it seems to be legitimate to carry out direct comparisons between both groups.

## Conclusions

The German version of the Social functioning Scale shows good obligatory psychometric properties, regarding internal consistency and validity. Significant correlations with the GAF Score, CGI score and with the DAS-M General score are indicating accordance with external criteria and observer ratings, respectively. Our data can be seen in line with previous findings concerning the original English version, the Spanish versions, and the Norwegian version of the Social Functioning Scale. The German version of the SFS represents a useful and practicable instrument for assessing social functioning and provides additional information to commonly used external assessment scales.
